# Characteristics of the retinal microvasculature in association with cardiovascular risk markers in children with overweight, obesity and morbid obesity

**DOI:** 10.1038/s41598-018-35279-6

**Published:** 2018-11-16

**Authors:** Jesse Rijks, Anita Vreugdenhil, Elke Dorenbos, Kylie Karnebeek, Peter Joris, Tos Berendschot, Ronald Mensink, Jogchum Plat

**Affiliations:** 1 Centre for Overweight Adolescent and Children’s Healthcare (COACH), Department of Paediatrics, Maastricht University Medical Centre, Maastricht, The Netherlands; 2 School of Nutrition and Translational Research in Metabolism (NUTRIM), Maastricht University, Maastricht, The Netherlands; 3 University Eye Clinic Maastricht, Maastricht University Medical Centre, Maastricht, The Netherlands; 4 Department of Human Biology, Maastricht University, Maastricht, The Netherlands

## Abstract

To aim of this study was to evaluate characteristics of the retinal microvasculature, but particularly potential associations with classic and novel (endothelial function and low-grade inflammation)markers for cardiovascular risk, in a cohort of children with overweight and (morbid) obesity. Central retinal arteriolar equivalent(CRAE) and central retinal venular equivalent(CRVE) were assessed. CRAE was significantly lower and AVR significantly higher in children with morbid obesity than in children with overweight and normal weight(p < 0.01). CRVE did not differ significantly between the four weight categories. A multiple linear regression model with CRAE as dependent variable showed that only DBP z-score(β = −2.848,p = 0.029) and plasma glucose concentrations(β = 6.029,p = 0.019) contributed significantly to the variation in CRAE. Remarkably, despite a correlation between CRAE and circulating concentrations of the adhesion molecules VCAM-1 or ICAM-1, markers for inflammation and endothelial function did not contribute to the variation in CRAE. This is the first study showing in population of children with overweight and obesity that the retinal arteriolar microvasculature, but not venular diameter is aberrant, with increasing BMI z-score. CRAE was significantly associated with several cardiovascular risk markers, and multiple linear regression showed that a higher diastolic blood pressure z-score and lower fasting plasma glucose concentrations significantly contributed to the variance in CRAE.

## Introduction

Children with overweight and obesity, and in particular with morbid obesity, have a high risk to develop cardiovascular disease, both during their youth and in adulthood^1,2^. Early detection of cardiovascular abnormalities is therefore of utmost importance for adequate risk assessment and initiation of targeted interventions. Various well-known factors including elevated lipid and lipoprotein concentrations, high blood pressure, and insulin resistance that contribute to low-grade inflammation and oxidative stress, and ultimately translate into endothelial dysfunction, are already present at a young age in children with overweight and obesity^1–4^. Endothelial dysfunction is considered as the earliest stage in the development of cardiovascular disease, which precedes clinical manifestation of symptoms^5–7^. Apparently, endothelial dysfunction develops in the microcirculation before affecting macrovascular structures^5–7^. A non-invasive method for early detection of microvascular derangements is evaluation of characteristics from the retinal microvasculature using fundus photography. In adults, narrower retinal arteriolar diameters and wider retinal venular diameters have been associated with increased cardiovascular risk^8–11^. Increasing evidence suggests that cardiovascular risk markers in early life are also associated with structural changes in the retinal microvasculature^12,13^. In population-wide cohort studies in children and adolescents, both retinal arteriolar and venular diameters have been associated with body mass index (BMI)^14–21^. Furthermore, associations between narrower retinal arteriolar diameters and increased blood pressure, and associations between wider venular diameters, increased triacylglycerol (TAG) and insulin concentrations have been demonstrated in children^15,16,18,22^. Although the retinal microvasculature has already been studied in several large population-wide cohort studies in children^14–20,22,23^, studies investigating characteristics of the retinal microvasculature and cardiovascular risk markers in the specific high CVD-risk group of children with overweight and (morbid) obesity are absent. Moreover, although there are numerous studies evaluating the association between retinal vasculature and classical CVD risk factors like blood pressure, serum lipids and insulin resistance, there is a lack of detailed knowledge regarding associations between the retinal microvasculature and markers reflecting inflammation and endothelial dysfunction. Regarding inflammation, scattered and inconsistent information regarding associations between retinal calibers and CRP can be found^12^, however associations with circulating pro-inflammatory cytokines and chemokines have to the best of our knowledge not been evaluated. In this cross-sectional study, we therefore aimed to evaluate characteristics of the microvasculature in the fundus in a selected high-risk group of children with overweight and (morbid) obesity and to evaluate associations with anthropometric parameters, cardiovascular risk markers, and markers for inflammation and endothelial function.

## Results

In total, 226 children (43% boys) with overweight, obesity or morbid obesity, a median age of 13.0 (4.5–18.9) years and a median BMI z score of 3.25 (1.17–5.28) were enrolled. Twenty percent (%) was classified as overweight (n = 46), 46% obese (n = 104), and 34% morbidly obese (n = 76). Baseline characteristics for the complete group and stratified by weight status category are presented in Table 1. In addition to these children we also included 15 healthy children (7 boys and 8 girls), aged 9.4 ± 1.1 years, with a normal body weight (BMI z score −1.41–1.18) and evaluated characteristics of their retinal vasculature as well. CRAE and AVR differed significantly between weight status categories (p = 0.000 and p = 0.002 respectively). For CRAE, post-hoc analyses showed significant differences between children with normal weight and overweight (p = 0.027), normal weight and obesity (p = 0.002), normal weight and morbid obesity (p = 0.000), overweight and morbid obesity (p = 0.007), and children with obesity and morbid obesity (p = 0.048; Table 2). For AVR, post-hoc analyses showed significant differences between children with normal weight and obesity (p = 0.037), normal weight and morbid obesity (p = 0.001), overweight and morbid obesity (p = 0.016), and children with obesity and morbid obesity (p = 0.011; Table 2). In contrast to the CRAE and AVR, the CRVE did not differ significantly between the weight status categories (Table 2). Several cardiovascular risk markers including serum total cholesterol (TC), LDL-cholesterol (LDL-C), HDL-cholesterol (HDL-C), C-reactive protein (CRP), interleukin 6 (IL-6), and insulin concentrations were significantly different between the three weight status categories, and increased across weight status categories, except for HDL-C that decreased across weight status categories (Table 3). Overall, children with morbid obesity had a more aberrant cardiovascular risk profile as compared to children with overweight and obesity (Table 3).

**Table 1:** Characteristics of the study participants stratified by weight status category.

	Total (n = 226)	Overweight (n = 46)	Obesity (n = 104)	Morbid obesity (n = 76)
Age	13.0 (4.5–18.9)^a^	12.2 (7.5–18.4)	12.2 (6.8–18.1)^d^	14.8 (4.5–18.9)^d^
Male/Female, %	43/57	33/67	47/53	43/57
Caucasian, %	77	87	76	74
BMI z score	3.25 (1.17–5.28)^b^	2.37 (1.17–2.92)^e^	3.14 (2.53–3.87)^e^	3.88 (3.37–5.28)^e^
Waist circumference z score	5.2 (1.7–13.0)^b^	3.6 (1.7–7.2)^e^	4.8 (2.3–9.3)^e^	7.0 (2.8–13.0)^e^
Hip circumference z score	4.0 (0.6–10.5)^b^	2.5 (0.8–5.1)^e^	3.7 (1.2–6.2)^e^	5.5 (0.6–10.5)^e^
Waist-to-hip ratio	0.91 (0.72–1.49)	0.9 (0.8–1.0)	0.9 (0.7–1.1)	0.9 (0.7–1.5)

Data presented as mean ± SD or as median (minimum-maximum). Children were classified as overweight, obese, or morbidly obese based on the International Obesity Task Force criteria^34^. ^a^Statistically different between the three weight status categories, p < 0.05; ^b^Statistically different between the three weight status categories, p < 0.01; ^c^Statistically different between children with overweight and children with morbid obesity, p < 0.0167; ^d^Statistically different between children with obesity and children with morbid obesity, p < 0.0167; ^e^Statistically different between all three w^e^ight status categories, p < 0.0167.

**Table 2:** Retinal calibers of the study participants stratified by weight status category

	Normal weight (n = 15)	Overweight (n = 46)	Obesity (n = 104)	Morbid obesity (n = 76)
CRAE, um	157.4 ± 14.4^a,b,c^	146.7 ± 14.7^d^	143.4 ± 17.0^e^	138.6 ± 16.0
CRVE, um	232.2 ± 10.3	228.5 ± 16.5	224.2 ± 22.8	226.9 ± 19.4
AVR	0.68 ± 0.06^b,c^	0.64 ± 0.07^d^	0.64 ± 0.07^e^	0.61 ± 0.07

Data presented as mean ± SD. Children were classified as normal weight, overweight, obese, or morbidly obese based on the International Obesity Task Force criteria^34^. ^a^Statistically different between children with normal weight and children with overweight (P < 0.05); ^b^Statistically different between children with normal weight and children with obesity, P < 0.05; ^c^Statistically different between children with normal weight and children with morbid obesity, P < 0.001; ^d^Statistically different between children with overweight and children with morbid obesity, P < 0.017; ^e^Statistically different between children with obesity and children with morbid obesity, P < 0.05.

**Table 3:** Cardiovascular risk markers stratified by weight status category.

	Total (n = 226*; **)	Overweight (n = 46*; **)	Obesity (n = 104 *; **)	Morbid obesity (n = 76*; **)
Total cholesterol, mmol/L	4.4 ± 0.8^a^	4.3 ± 0.8^c^	4.4 ± 0.8^d^	4.7 ± 0.8^c,d^
LDL-cholesterol, mmol/L	2.7 ± 0.7^b^	2.4 ± 0.7^c^	2.6 ± 0.7^d^	2.9 ± 0.7^c,d^
HDL-cholesterol, mmol/L	1.2 (0.5–2.6)^b^	1.4 (0.8–2.6)^c^	1.3 (0.6–2.1)^d^	1.1 (0.5–1.8)^c,d^
Triacylglycerol, mmol/L	1.04 (0.39–4.75)^a^	0.95 (0.39–2.85)^c^	1.03 (0.39–2.74)	1.14 (0.42–4.75)^c^
Free-fatty acids, mmol/L	0.64 (0.23–1.28)	0.62 (0.23–0.98)	0.66 (0.25–1.20)	0.60 (0.28–1.28)
C-reactive protein, mg/L	2.0 (1.0–38.0)^b^	2.0 (1.0–7.0)^c^	2.0 (1.0–38.0)	4.0 (1.0–14.0)^c^
MCP-1, pg/mL	127.6 (71.9–459.5)	114.8 (83.4–396.5)	129.1 (71.9–344.1)	133.4 (74.0–459.5)
SAA, μg/mL	2.4 (0.1–45.5)	2.0 (0.4–8.6)	2.1 (0.1–45.5)	3.8 (0.6–21.0)
IL-6, pg/mL	0.76 (0.00–3.66)^a^	0.67 (0.32–1.42)^c^	0.72 (0.19–3.66)	0.97 (0.00–2.83)^c^
IL-8, pg/mL	2.76 (0.88–21.42)	2.86 (1.85–17.93)	2.86 (0.88–17.32)	2.40 (1.03–21.42)
E-selectin, ng/mL	15.3 (1.7–45.1)	15.3 (4.0–45.1)	15.2 (4.9–40.1)	15.5 (1.7–36.7)
ICAM-1, μg/mL	469 (235–911)	475 (304–742)	474 (235–911)	457 (302–676)
VCAM-1, μg/mL	724 (453–1324)	735 (530–1241)	762 (453–1324)	672 (471–981)
Systolic blood pressure z score	0.35 ± 1.11^b^	−0.05 ± 1.11^c^	0.29 ± 1.06	0.67 ± 1.10^c^
Diastolic blood pressure z score	−0.50 ± 1.14^a^	−0.78 ± 1.01^c^	−0.60 ± 1.12	−0.19 ± 1.17^c^
Plasma glucose, mmol/L	4.1 (2.5–5.9)	4.1 (3.1–5.2)	4.1 (2.8–5.8)	4.2 (2.5–5.9)
Insulin, mU/L	15.7 (2.0–158.0)^b^	10.9 (2.0–30.3)^c^	14.0 (2.4–111.2)^d^	22.2 (5.1–158.0)^c,d^
HOMA-IR	2.75 (0.43–25.98)^b^	2.11 (0.43–5.12)^c^	2.62 (0.43–19.27)^d^	4.00 (0.94–25.98)^c,d^
HbA1c, %	5.2 (4.1–6.1)	5.2 (4.5–6.1)	5.2 (4.1–6.0)	5.3 (4.4–6.1)
Median sensor glucose, mmol/L	5.0 (2.7–7.3)	4.7 (2.7–6.9)	5.0 (3.7–7.3)	5.1 (3.6–6.0)
Maximum sensor glucose, mmol/L	7.0 (5.6–10.8)	7.4 (5.6–10.2)	6.9 (5.7–10.8)	7.4 (5.6–9.0)
Minimum sensor glucose, mmol/L	3.4 (2.2–5.1)	3.6 (2.2–4.3)	3.2 (2.3–4.6)	3.5 (2.2–5.1)
CONGA1	0.58 (0.28–1.09)	0.62 (0.32–1.09)	0.57 (0.28–1.06)	0.58 (0.31–0.90)
CONGA2	0.72 (0.31–1.62)	0.78 (0.36–1.62)	0.72 (0.31–1.36)	0.72 (0.39–0.99)
CONGA4	0.85 (0.35–2.06)	0.89 (0.45–2.06)	0.79 (0.35–1.72)	0.88 (0.43–1.24)

Data presented as mean ± SD or as median (minimum-maximum). Children were classified as overweight, obese, or morbidly obese based on the International Obesity Task Force criteria^34^. *MCP-1, SAA, IL-6, IL-8, E-selectin, ICAM-1, and VCAM-1 were measured in a subgroup (total group n = 170; overweight n = 33; obesity n = 82; morbid obesity n = 55); **Sensor glucose concentrations and CONGA were measured in a subgroup (total group n = 73; overweight n = 14; obesity n = 31; morbid obesity n = 28). ^a^Statistically different between the three weight status categories, p < 0.05; ^b^Statistically different between the three weight status categories, p < 0.01; ^c^Statistically different between children with overweight and children with morbid obesity, p < 0.0167. ^d^Statistically different between children with obesity and children with morbid obesity, p < 0.0167. MCP-1 = monocyte chemoattractant protein 1; SAA = serum amyloid A protein; IL-6 = interleukin 6; IL-8 = interleukin 8; ICAM-1 = intracellular adhesion molecule 1; VCAM-1 = vascular cell adhesion protein 1; HOMA-IR = Homeostatic Model Assessment of Insulin Resistance; CONGA = Continuous Overlapping Net Glycaemic Action; presented for 1, 2, or 4 h time differences.

### Arteriolar retinal vessel diameter and associations with cardiovascular risk markers

CRAE stratified for anthropometric characteristic quartiles and cardiovascular risk parameter quartiles are presented in Supplementary Fig. 1. A significant negative p for trend was found for CRAE between BMI z score quartiles (p = 0.008), waist- and hip circumference z score quartiles (p = 0.006; p = 0.009, respectively), serum TC concentration quartiles (p = 0.0382), serum LDL-C concentration quartiles (p = 0.001), and systolic and diastolic blood pressure quartiles (p = 0.009; p = 0.005, respectively) (Supplementary Fig. 1). A significant positive p for trend was found for serum intracellular adhesion molecule 1 (ICAM-1) concentration quartiles (p = 0.031) and serum vascular cell adhesion molecule 1 (VCAM-1) concentration quartiles (p = 0.003) (Supplementary Fig. 1).

Furthermore, the positive p for trend for plasma glucose concentrations quartiles nearly reached significance (p = 0.054) (Supplementary Fig. 1). In contrast to plasma glucose concentrations, no associations were found between CRAE and sensor glucose concentrations or the continuous overlapping net glycaemic action (CONGA).

Next, BMI z score and cardiovascular risk markers with a significant or nearly significant p for trend were entered into one multiple linear regression model with CRAE as dependent variable. Altogether, this model explained 15.3% of the variance in CRAE, in which diastolic blood pressure (DBP) z score (β = −2.848, p = 0.029) and plasma glucose concentrations (β = 6.029, p = 0.019) contributed significantly (Table 4) Results were the same regardless of whether age or gender was added to the prediction model.

**Table 4:** Regression analysis for central retinal arteriolar and retinal venular equivalent.

	CRAE				CRVE		
	(μm per unit change)				(μm per unit change)		
	β (95% CI)	p-value	R^2^		β (95% CI)	p-value	R^2^
BMI z score	−2.489 (−6.037–1.059)	0.168	0.153	*BMI z score*	−2.814 (−7.198–1.570)	0.207	0.028
Plasma glucose, mmol/L	6.029 (1.016–11.042)	0.019		*C-reactive protein*, *mg/L*	0.674 (−0.252–1.599)	0.152	
LDL-cholesterol, mmol/L	−2.913 (−6.234–0.408)	0.085		*HOMA-IR*	0.701 (−0.197–1.598)	0.125	
ICAM-1, μg/mL	0.019 (−0.007–0.045)	0.149		*HbA1c*, *%*	0.213 (−9.005–9.430)	0.964	
VCAM-1, μg/mL	0.006 (−0.013–0.025)	0.509					
Systolic blood pressure z score	−0.420 (−3.136–2.296)	0.760					
Diastolic blood pressure z score	−2.848 (−5.400–0.296)	0.029					

Data represented as unstandardized regression coefficient (95% CI); CRAE = central retinal arteriolar equivalent; CRVE = central retinal venular equivalent; ICAM-1 = intracellular adhesion molecule 1; VCAM-1 = vascular adhesion protein 1; HOMA-IR = Homeostatic Model Assessment of Insulin Resistance.

### Venular retinal vessel diameters and associations with cardiovascular risk markers

CRVE stratified for anthropometric characteristic quartiles and cardiovascular risk parameter quartiles are presented in Supplementary Fig. 2. A significant p for trend was found for CRVE and serum CRP concentration quartiles (p = 0.049), homeostatic model assessment of insulin resistance (HOMA-IR) quartiles (p = 0.040), and serum HbA1c concentration quartiles (p = 0.031) (Supplementary Fig. 2). No associations were found between CRVE and sensor glucose concentrations or the CONGA.

BMI z score and cardiovascular risk markers with a significant p for trend were entered into one multiple linear regression model with CRVE as dependent variable. However, none of the markers was significantly associated with CRVE (Table 4). Results were the same regardless of whether age or gender was added to the prediction model.

## Discussion

We here show in a large group of high CVD risk children with overweight, and (morbid) obesity that severity of overweight within this risk group is associated with arteriolar but not venular retinal microvasculature. Also in comparison to retinal calibre data from healthy children with a normal weight only the CRAE shows differences with children with overweight, obesity and morbid obesity but not the CRVE. Our results extend the results of previous studies in samples of general population-wide approaches, reporting an association between the CRAE and BMI^14–21^. Compared to these cohort studies, the calculated CRAE seems notably narrower and the CRVE wider in our high CVD risk study population of children with overweight and (morbid) obesity. This may at least partly be due to their increased body weight and concomitant metabolic disturbances. Indeed, in our study the CRAE was even 8.1 µm narrower in children with morbid obesity as compared to children with overweight. In addition, children with morbid obesity had a more adverse cardiovascular risk profile. P for trend analyses showed that CRAE was significantly associated with several cardiovascular risk markers, including serum TC, LDL-C and glucose concentrations, and systolic blood pressure (SBP) and DBP z scores. These results suggest that the disturbed metabolic profiles in children with morbid obesity are not only associated with alterations in macrovascular risk markers^1–4^, but also in microvascular risk markers. Interestingly, Tirsi *et al*.^21^ showed in adolescents, in line with our data, that arteriolar diameters were associated with BMI, but also that CRAE associated with measures of brain health. Smaller retinal arteriole diameters were associated with more global cerebral atrophy and smaller hippocampal volumes. This illustrates that the fundus characteristics which we here show, and which are easily accessible in children are already clearly deviating at a very young age and can be of great clinical value, not only in the context of risk prediction of peripheral CVD but probably also for other areas such as cerebral microvascular disease.

Multiple linear regression analysis showed that DBP z score and fasting plasma glucose concentrations contributed significantly to CRAE in children with overweight and (morbid) obesity. Interestingly, fasting plasma glucose concentrations were positively related to CRAE rather than negative as was a priori expected. Studies investigating the relation between fasting plasma glucose concentrations and retinal microvasculature characteristics in children in general are limited. To the best of our knowledge, so far only Hanssen *et al*. evaluated this association and found no relationship between fasting plasma glucose concentrations and retinal microvasculature in a sample of the general paediatric population^15^. In children with type 1 diabetes, however, wider arteriolar vessels predicted development of retinopathy^24,25^, and several studies in adults demonstrated that the CRAE was significantly wider in participants with type 2 diabetes mellitus as compared to non-diabetic participants^26–29^. Altogether these findings suggest that higher glucose concentrations are related to wider arterioles, which is associated with lower CVD risk. The underlying mechanism for this unexpected positive direction between plasma glucose concentrations and CRAE is not yet understood, but it has been postulated that hyperglycaemia initiates retinal dilation through hyperperfusion and impaired auto regulation^30,31^. However, it should be emphasized that plasma glucose concentrations in our study were within high-normal ranges in the vast majority of the children. In addition, arteriolar and venular diameters were not associated with the sensor glucose concentrations or glycaemic variability in free-living conditions. Future studies in children with overweight and (morbid) obesity are required to further investigate underlying mechanisms of the association between fasting glucose concentrations and retinal microvasculature.

DBP z score was negatively related to CRAE. In line with our findings, previously cohort studies in children also demonstrated an association between a narrower CRAE and higher DBP^15,18,22,23^. Recently, The Young Finns Study demonstrated that high blood pressure in childhood and increased blood pressure from childhood to adulthood affects retinal microvasculature, suggesting that cardiovascular disease risk origins in early life^32^. Together with our findings, this highlights the importance of early recognition of young children with overweight and (morbid) obesity for adequate risk assessment and intervention. Additionally, these findings stress the urgency for lifestyle intervention studies with long-term follow up, to investigate whether lifestyle improvement translates into improvement of retinal vessel diameter and reduced cardiovascular disease risk in children with overweight and (morbid) obesity.

It is generally accepted that endothelial dysfunction is the earliest stage in the development of cardiovascular disease, which largely precedes clinical manifestation of symptoms. This endothelial dysfunction develops in the microcirculation before affecting macrovascular structures^5–7^. Therefore, the correlation that we found between s-ICAM and s-VCAM, both markers reflecting activation of the endothelium and established markers for endothelial dysfunction, and characteristics of the microcirculation in the fundus could be expected. It was however remarkable that the correlations could only be shown for CRAE and not for CRVE. Apparently endothelial dysfunction in children with overweight and obesity predominantly occurs in the arterioles, potentially as a consequence of the metabolic disturbances since also the dyslipidemia together with the hypertension profile showed these correlations. Clearly this association between blood pressure, metabolic dysregulation and CRAE was not linked to circulating cytokines and chemokines. Therefore it might be that inflammation is not the predominant trigger for the metabolic induced aberrations in CRAE. In the multiple regression analysis the endothelial function parameters lost their significant contribution illustrating that metabolic and blood pressure parameters are quantitatively more important.

Interestingly, while CRAE was significantly different between overweight status categories, CRVE was comparable between the categories. This suggests that different physiological processes affect the retinal arteriolar and venular microvasculature. P for trend analyses showed that CRVE was significantly associated with HOMA-IR, and HbA1c and CRP concentrations. For CRP, results are in line with those of previous cohort studies in children^14,15^. However, CRP concentrations in our study did not contribute significantly to the CRVE in multivariate regression analysis. It is well known that various additional factors also have an influence on the retinal vascular caliber. For example, ethnicity, dietary factors and exercise have all been shown to influence retinal vascular caliber^12^. It is evident that including these factors in the multiple regression models would have increased the explained variance of the model, but unfortunately we did not have this data accessible.

## Conclusion

In conclusion, the arteriolar retinal microvasculature is already aberrant at a young age in a high CVD risk population of children with overweight, obesity, and especially morbid obesity. Specific cardiovascular risk markers including serum TC, LDL-C, glucose, ICAM-1, VCAM-1 concentrations, as-well-as SBP and DBP z scores are associated with the arteriolar retinal diameter. Interestingly, also the endothelial dysfunction markers ICAM and VCAM correlated with CRAE. However in a multivariate regression approach, only higher DBP z scores and lower fasting plasma glucose concentrations remained to contribute significantly to a narrower retinal arteriolar diameter. Long-term longitudinal follow-up studies are necessary to investigate whether lifestyle improvement translate into improvement of retinal vessel diameter in children with overweight and (morbid) obesity.

## Methods

### Setting

This study was designed and conducted within the setting of the Centre for Overweight Adolescent and Children’s Healthcare (COACH) at the Maastricht University Medical Centre (Maastricht UMC+). Within COACH, the health status of children with overweight and (morbid) obesity was evaluated, and they were monitored and guided as described previously^3^. Briefly, participation in the COACH program commenced with a comprehensive assessment to exclude underlying syndromic or endocrine conditions of their increased body weight, to evaluate complications and risk factors associated with overweight and (morbid) obesity, and to obtain insight into behaviour and (family) functioning. The assessment included, amongst others, fasting blood examination and fundus photography. After the assessment, all children and their families were offered on-going, tailored, and individual guidance with a focus on lifestyle changes with regular visits at the outpatient clinic. By focusing on small, step-by-step lifestyle improvements, the program aimed to convert the lifestyle changes to daily habits^3^.

### Study participants

Children who started participating in the COACH program between 2011 and 2015 and from whom fundus images were available at the start of their participation were retrospectively included. The presence of diabetes mellitus was an exclusion criteria for participation in this study, since changes in microvasculature are a well-known complication of diabetes mellitus^33^. Finally, 226 children with overweight, obesity or morbid obesity were eligible for inclusion. From another study in school-based children we obtained data from 15 healthy children with a normal body weight. The studies were conducted according the guidelines administered by the Declaration of Helsinki and approved by the medical ethical committee of the Maastricht UMC + . Informed consent was obtained from a parent and/or legal guardian for study participation before the start of the measurements.

### Anthropometric characteristics

Anthropometric data were acquired while children were barefoot and wearing only underwear. Body weight was determined using a digital scale (Seca) and body length was measured using a digital stadiometer. BMI was calculated and BMI z scores were obtained using a growth analyser (Growth Analyser VE). The BMI z score reflects a measure of weight, adjusted for height, sex, and age. Based on the International Obesity Task Force criteria children were classified as overweight, obese, or morbidly obese^34^. Waist circumference was measured with a non-elastic tape at the end of a natural breath at midpoint between the top of the iliac crest and the lower margin of the last palpable rib. Hip circumference was measured at the widest portion of the buttocks. Waist- and hip circumference z scores were determined^35^, waist-to-hip ratio (WHR) was calculated, and ethnicity was defined^36^.

### Retinal microvasculature assessment

Retinal vascular images were made to assess microvascular diameters in the right eye with a retina camera (TRC-NW300; Topcon Co; Tokyo; Japan), while the children were seated with their chin placed on a chin rest and their forehead against a bar to keep their heads steady. The digital image analysis software Vasculo-matic ala Nicola (IVAN; Department of Ophthalmology and Visual Science; University of Wisconsin-Madison; Madison; USA) was used to analyse the photographs. IVAN automatically detected the blood vessels of an image and the researcher subsequently distinguished between arterioles from venules, and selected at least three arterioles and three venules coursing through an area 0.5 to 1 disc diameter from the optic disc margin. Vessel diameters were calculated according the improved Parr Hubbard (PH) formula^37^ which resulted in the calculation of the CRAE and CRVE.

### Cardiovascular risk markers

In all children, a fasting lipid and lipoprotein profile including serum TC, LDL-C, HDL-C, TAG, and free fatty acids (FFA) concentrations, was measured (Cobas 8000 modular analyser, Roche). Further, fasting plasma glucose (Cobas 8000 modular analyzer, Roche), serum insulin (Immulite-1000, Siemens Healthcare Diagnostics), and HbA1c concentrations (HPLC Variant II, Bio-Rad Laboratories) were measured. Insulin resistance was estimated using the HOMA-IR^25^. HOMA-IR is a simple, inexpensive substitute for insulin resistance derived from a mathematical assessment of the balance between hepatic glucose output and insulin secretion, for which only fasting plasma glucose and fasting serum insulin are required. The following formula was applied: fasting glucose (mmol/L) x fasting insulin (µU/L)/22.5^38^. Daytime SBP and DBP was measured about 20 times during a period of 1.5 hours with an interval of three minutes between each measurement using the Mobil-O-Graph (I.E.M. GmbH). Mean BP was calculated using these 20 measurements. The size of the cuff used corresponded with the circumference of the upper arm. SBP and DBP z scores were calculated according to reference values related to height and sex^39^. In a randomly selected subgroup (n = 170) of the participating children, a panel of markers reflecting pro-inflammatory status (monocyte chemoattractant protein 1(MCP-1), serum amyloid A protein (SAA), IL-6, and interleukin 8 (IL-8)), and endothelial dysfunction (VCAM-1, ICAM-1, and E-selectin) were measured (Multi Spot ELISA assay, Meso Scale Discovery). Furthermore, glucose concentrations were measured in free-living conditions using a continuous glucose-monitoring (CGM) sensor for 48-hours, again in a subgroup of children (n = 73), as described previously^40^. Median, minimum, and maximum sensor glucose concentrations were calculated, and the intra-day glycaemic variability, which reflects acute glucose fluctuations, was assessed by the CONGA^41^. In this study CONGA1, CONGA2, and CONGA4 were used based on 1, 2 and 4-hour time differences, respectively. In essence, these time differences corresponded approximately to time between different activities in school, time between snacks, and time between meals.

### Statistical analysis

All statistical analyses were performed using SPSS 23.0 for Windows (SPSS Inc). Shapiro-Wilk test was performed to test for normality. Differences in baseline characteristics between groups were analysed with a *X*^2^-test, one-way analysis of variance (ANOVA), or Kruskal-Wallis test, as appropriate. If there was a significant difference between groups, post-hoc tests using the least significance difference (LSD) method or the Mann-Witney *U* test were conducted, as appropriate. Anthropometric characteristics and cardiovascular risk markers were divided into quartiles. P for trend was calculated between quartiles. Relationships between variables were determined by multiple linear regressions models. A *p*-value below 0.05 was considered statistically significant. Data are presented as means with standard deviations or as medians with the minimums and maximums.

### Clinical trial registration

Clinical trial registration at ClinicalTrial.gov; Registration Number: NCT02113644. First Posted: April 14, 2014. Last Update Posted: August 31, 2017.

Clinical trial registration at ClinicalTrial.gov; Registration Number: NCT03440580. First Posted: February 21, 2018. Last Update Posted: August 20, 2018.

## Electronic supplementary material

Supplementary figures

## Data Availability

The datasets generated during and/or analysed during the current study are available from the corresponding author on reasonable request.
