# Supplementary material for: Characteristics of the retinal microvasculature in association with cardiovascular risk markers in children with overweight, obesity and morbid obesity
Source: Sci Rep. 2018 Nov 16;8:16952. doi: 10.1038/s41598-018-35279-6 (PMC6240121; doi:10.1038/s41598-018-35279-6)
Supplement: Supplementary file 1 — Supplementary figures [file 41598_2018_35279_MOESM1_ESM.pdf]

Characteristics of the retinal microvasculature in association with cardiovascular risk markers in children with overweight, obesity and morbid obesity

Jesse Rijks[a,b] ; Anita Vreugdenhil[a,b *]; Elke Dorenbos[a,b] ; Kylie Karnebeek[a,b]; Peter Joris[b,d];
Tos Berendschot[c;] Ronald Mensink[b,d] ; Jogchum Plat[b,d]

a. Centre for Overweight Adolescent and Children's Healthcare (COACH), Department of Paediatrics, Maastricht University Medical Centre, Maastricht, The Netherlands

b. School of Nutrition and Translational Research in Metabolism (NUTRIM), Maastricht University, Maastricht, The Netherlands

c. University Eye Clinic Maastricht, Maastricht University Medical Centre, The Netherlands.

d. Department of Human Biology, Maastricht University, Maastricht, The Netherlands

Supplementary Information

Supplementary Figure 1. Central retinal arteriolar equivalent stratified for anthropometric characteristic quartiles and cardiovascular risk parameter quartiles with a significant p for trend

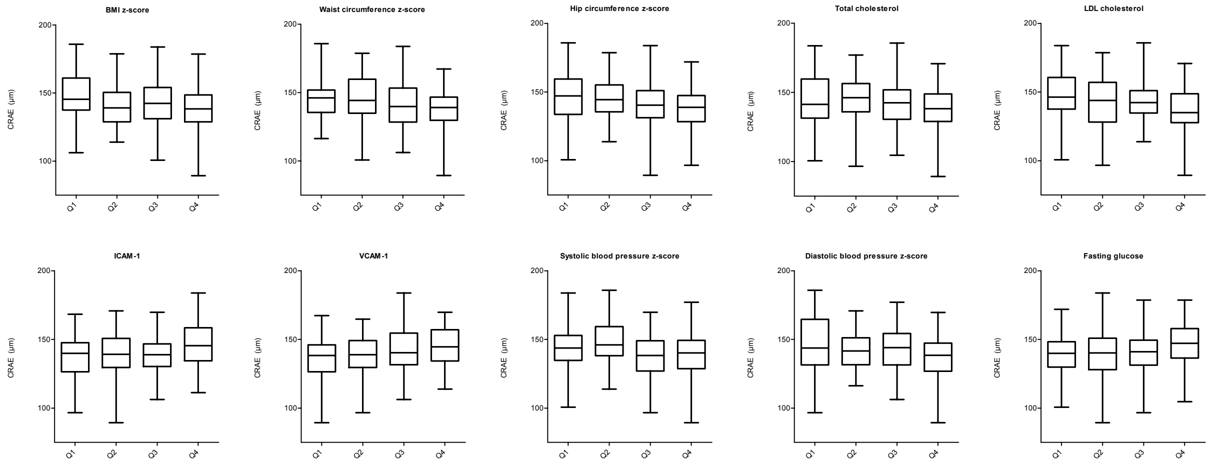

Data presented as mean with minimum and maximum. P for trends: BMI z score p=0.008; waist circumference z score p=0.006; hip circumference z score p=0.009; serum total cholesterol concentrations p=0.038; serum LDL cholesterol concentrations p=0.001; serum ICAM-1 concentrations p=0.031; serum VCAM-1 concentrations p=0.003; systolic blood pressure z score p=0.009; diastolic blood pressure z score p=0.005; plasma glucose concentrations p=0.054. CRAE = central retinal arteriolar equivalent; ICAM-1= intracellular adhesion molecule 1; VCAM-1=vascular cell adhesion molecule 1

Supplementary Figure 2. Central retinal venular equivalent stratified for cardiovascular risk parameter quartiles with a significant p for trend

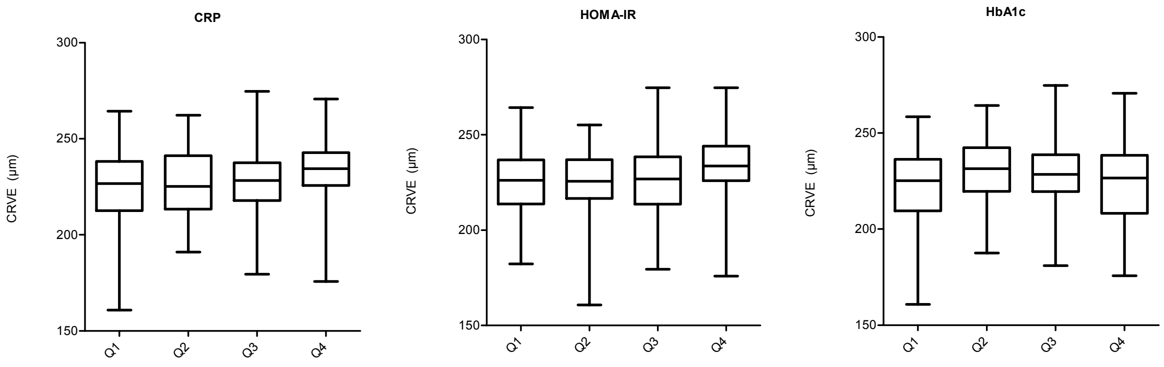

Data presented as mean with minimum and maximum. P for trends: serum CRP concentrations p=0.049; HOMA-IR p=0.040; serum HbA1c concentrations p=0.031. CRVE=central retinal venular equivalent; HOMA-IR = Homeostatic Model Assessment of Insulin Resistance;
